# Modulating the Bioavailability and Bioaccessibility of Polyphenolic Compounds and Enhancing Health-Promoting Properties Through the Addition of Herbal Extracts to a Functional Beverage

**DOI:** 10.3390/molecules30244796

**Published:** 2025-12-16

**Authors:** Hanna Mikołajczak, Paulina Nowicka

**Affiliations:** Department of Fruit, Vegetables and Plant Nutraceutical Technology, Faculty of Biotechnology and Food Science, Wrocław University of Environmental and Life Sciences, 51-360 Wrocław, Poland; 121632@student.upwr.edu.pl

**Keywords:** boosted beverages, polyphenolic compounds, anti-inflammatory effect, antioxidant activity, sensory evaluation

## Abstract

Shots are becoming increasingly popular due to their convenience and concentrated nutrient content. In this study, innovative shots were developed as herbal-enriched formulations designed to improve bioaccessibility, bioavailability, and health-promoting properties. To achieve this, pear–flowering quince juice was enriched with a mixture of herbal infusions and evaluated for its physicochemical characteristics, including bioactive compounds, as well as its functional and sensory properties. Additionally, the products were subjected to a three-stage in vitro digestion model (oral–gastric–small intestine) to assess bioaccessibility and bioavailability. The results revealed that the shot containing mint and nettle had the highest polyphenolic content (579 mg/100 mL), while the shot enriched with white mulberry and common yarrow had the highest mineral content (28 mg/100 mL). The developed formulations also exhibited strong inhibitory effects on pancreatic lipase and lipoxygenase. It was demonstrated that the addition of selected herbs, particularly those rich in rosmarinic acid, can enhance both bioaccessibility and bioavailability, and that menthol may further potentiate these effects. In conclusion, the study showed that incorporating different types of herbs into pear–flowering quince juice enables the development of novel products with tailored health-promoting and sensory properties, primarily through the synergistic action of the individual ingredients.

## 1. Introduction

In recent years, the impact of dietary habits and the quality of consumed products on human health and well-being has become a frequent topic of public debate. Consumer awareness of healthy eating is steadily increasing, with people paying attention not only to the number of calories they consume but also to the quality of their diet. This shift is a result of changes in eating habits and lifestyle, as consumers increasingly focus on the health benefits of food. However, a significant portion of society still adheres to an Ultra-processed food intake, characterized by a high intake of fast food, ready-made meals, snacks, and sugary drinks. Such foods have a high glycemic index, which contributes to rapid spikes in blood glucose levels. Products rich in simple sugars, saturated fats, and salt may promote inflammation, exacerbate existing conditions, and contribute to obesity and heart disease [1,2]. Long-term consumption of an Ultra-processed food intake can contribute to pathological changes in lipid metabolism and energy balance in the human body, as well as negatively affect the activity of the immune system. It is believed that specific immune responses and metabolic regulation are closely interconnected, and their proper functioning depends on the interaction between these processes. Moreover, diet is a key factor in modulating the course of the inflammatory response, as its composition can either exacerbate or alleviate inflammation. Chronic activation of the immune system promotes the development of metabolic disorders such as obesity, atherosclerosis, and nonalcoholic fatty liver disease [3]. The harmful effects of Ultra-processed food intake on the body are also associated with the presence of free radicals in consumed food. These are atoms or ions with an unpaired electron, formed during processes such as frying and smoking. Their interaction with cellular structures—including nucleic acids, proteins, lipids, and carbohydrates—causes various types of damage, such as mutations, DNA strand breaks, and cell death [4].

Free radicals associated with Ultra-processed food intake could be counteracted by the use of medicinal and culinary herbs due to their content of bioactive compounds with a wide range of health-promoting properties, particularly their antioxidant capacity. Among them, the following should be mentioned: lemon balm, field horsetail, nettle, peppermint, small-leaved lime, hops, white mulberry and common yarrow. Lemon balm (*Melissa officinalis* L.) contains flavonoids and phenolic acids, while its essential oil is rich in citral and geranial, which provide calming and antibacterial effects [5]. Field horsetail (*Equisetum arvense* L.) supplies easily absorbable silica along with polyphenols, tannins, and flavonoids, supporting the health of skin, hair, and nails, and exhibiting antioxidant properties [6]. Nettle (*Urtica dioica* L.) is abundant in phenolic acids, chlorophylls, carotenoids, and unsaturated fatty acids, contributing to its anti-inflammatory and antioxidant properties [7]. Peppermint (*Mentha piperita* L.) contains menthol, phenolic acids, and flavonoids, giving it antibacterial, antinausea, and digestive-supporting effects [8]. Small-leaved lime (*Tilia cordata* Mill.) is a source of flavonoids, including quercetin and kaempferol, which neutralize free radicals and support antioxidant activity [9]. Hops (*Humulus lupulus* L.) are valued for unique compounds such as humulones and lupulones. In particular, xanthohumol and 6-prenylnaringenin and 8-prenylnaringenin have been identified as chemopreventive agents with potential activity against cancer development [10]. White mulberry (*Morus alba* L.) contains rutin, kaempferol, and the alkaloid DNJ, which provide antidiabetic and antioxidant effects [11]. Common yarrow (*Achillea millefolium* L.) is rich in flavonoids, phenolic acids, sesquiterpene lactones, and essential oils, demonstrating anti-inflammatory, antimicrobial, antioxidant, and antihyperglycemic properties [12]. The unique composition of these herbs, along with their wide range of health-promoting properties, makes them suitable for incorporation into food product development, including functional beverages, as donors of bioactive compounds and as modulators of sensory properties.

The growing awareness of healthy lifestyles among consumers has led to a rapid increase in the popularity of functional foods, including beverages enriched with active ingredients. Manufacturers are increasingly introducing functional drinks into their product lines, offering convenient solutions tailored to meet the fast-paced lifestyles typical of Western societies [13]. These products not only support the body but also promote well-being and help reduce the risk of disease. Functional beverages represent a relatively simple food matrix, which may facilitate the efficient release and absorption of their constituents, including electrolytes, sugars, and compounds with potential health-promoting properties. Due to their light form, such beverages can effectively deliver selected health-promoting compounds without placing excessive strain on the digestive system, although their absorption is modulated by factors such as chemical structure, solubility, and interactions between co-occurring compounds within the formulation [14].

Within this category, functional “shot” beverages (small-volume, high-concentration formulations) have experienced particularly dynamic growth [15]. These products typically contain elevated amounts of physiologically active compounds, including polyphenols, adaptogens, vitamins, caffeine, and botanical extracts, enabling consumers to obtain a meaningful dose of bioactive substances in a single, easy-to-consume serving. Their compact format enhances convenience, portability, and rapid intake, which aligns well with the current market demand for on-the-go functional nutrition. Scientific reports indicate that such concentrated beverages can effectively deliver antioxidant, anti-inflammatory, or energizing compounds, making them attractive to health-conscious consumers seeking targeted physiological benefits (e.g., immune support, cognitive enhancement, fatigue reduction) [16,17].

Therefore, the aim of this study was to develop a product that not only meets consumer expectations but also contributes to preventive health care through composition modulations. The following research hypothesis was formulated: the use of plant-based ingredients with scientifically proven anti-inflammatory and antioxidant properties may modulate the health-promoting properties of the product, the profile of its bioactive compounds, as well as their bioaccessibility and bioavailability. Therefore, the study not only developed formulations with health-promoting properties, but also examined a number of relationships between the individual components of the matrix and their impact on the degree of bioavailability of polyphenolic compounds. Accordingly, in this study, a mixture of flowering quince (*Chaenomeles japonica*) and pear (*Pyrus communis*) juices was used as the base, with herbal mixtures added. *Chaenomeles* fruit is valued for its high antioxidant activity, the presence of phenolic compounds, and its immune-supporting effects. Owing to its natural sweetness, pear enhances the drink’s palatability while also providing valuable compounds such as polyphenols. The consumption of pears has been associated with the prevention of cardiovascular diseases and type II diabetes [18,19].

Such products in this form have not been developed to date, and it was assumed that combining a fruit base with herbal extracts would enhance the pro-health effects of the resulting mixtures by fortifying the base with bioactive compounds of differing structures and properties, thereby resulting in a synergistic action. The development of this type of formulation holds the potential to deliver both health and economic benefits—an especially significant advantage in today’s world, where there is an urgent need to prevent chronic noncommunicable diseases.

## 2. Results and Discussion

### 2.1. Physicochemical Properties of Obtained Functional Beverages

A summary of all obtained products is presented in Table 1. In turn, the physicochemical parameters (color, turbidity stability, dry matter, soluble solids, total acidity, pH, osmosis, energy value, vitamin C, sugar content, and minerals) were evaluated in all obtained products immediately after processing (Table 2, Table 3 and Table 4).

The colors of the shot beverages were measured using the CIEL*a*b* system (Table 2). The L* parameter values in the evaluated products ranged from 45.31 to 51.62. The highest value of this parameter was observed in shot 0 and shot 3 (L* = 51.62), while the lowest value was found in shot 2 (L* = 45.31). Generally, it was observed that the addition of herbs made the beverage lighter due to a dilution effect caused by the additional volume of infusions added to the base. The a* parameter values ranged from −3.52 to 0.28. The most intense red color was observed in shot 2, which was the only one to show a positive a* value. The lowest a* values were observed in shot 0 (−3.52) and shot 3 (−3.4). Generally, it was noted that the addition of herbs made the beverage more red, with the exception of shot 3 (−3.4). The b* parameter values ranged from 13.46 to 18.10. The most intense yellow color was detected in shot 1 (b* = 18.10), while the lowest b* value was observed in shot 0 (b* = 13.46). This parameter continued to change with the addition of herbs, which caused a significant increase in the b* parameter. Furthermore, the calculated Chroma (C*) values ranged from 13.91 (100% juice) to 18.12 (product 1), indicating differences in color saturation between the formulations, with a general tendency for the addition of herbal infusions to increase Chroma. The hue angle (h°) ranged from approximately 89° (product 1) to 105° (100% juice), confirming that all beverages fell within the yellow–green region of the color space, typical of pome-based juices and herbal infusions. The different colors observed in the analyzed products are related to the specific characteristics of the added herbal materials or fruit components. For instance, flowering quince, known for its bright yellow skin and flesh, may contribute to a lighter coloration [20]. The darkest color, observed in shot 2, may result from the high content of various bioactive compounds in nettle, which undergo transformations when exposed to heat and oxygen—such as the change of chlorophyll into the darker, more olive-toned pheophytin, the oxidation of phenolic acids and flavonols, or the presence of iron and magnesium, which can alter the infusion’s color to a dull green [21]. In turn, the greenish appearance of ingredients like lime and hops corresponds to their natural pigmentation.

The high turbidity stability of the product ensures the absence of sedimentation of suspended raw material particles during storage. This parameter is important both from a technological standpoint and in terms of consumer desirability. However, a turbidity stability of at least 50% at 250 NTU is required to classify juices as cloudy [22]. The most stable were shots 1 and 2, with turbidity changes of 5.77% and 4.35% NTU, respectively. In contrast, shot 4 exhibited the lowest stability, with a turbidity change of 9.65% NTU. Despite this, all the analyzed products had turbidity stability below 50%, which is considered insufficient to classify them as cloudy.

Due to the liquid consistency of the tested beverages, the dry matter content remained at a low level (10.87–12.51%). The decrease in dry matter observed in the samples following the addition of herbal infusions is a result of dilution caused by incorporating an additional volume of infusions into the base. The highest dry matter content was recorded in shot 0 (12.51%), while the lowest values were observed in shots 2 and 4 (10.98% and 10.87%, respectively). The relatively low dry matter content across all formulations is characteristic of liquid beverages and confirms that the products remained within the expected physicochemical range. Although the incorporation of herbal infusions led to a significant reduction in this parameter, the resulting values still fell within the typical limits for liquid formulations, indicating that the applied infusion volume did not substantially compromise product stability, uniformity, or overall technological quality [23]. The stability of products with the addition of herbal infusions is also indicated by the extract results. Total extract refers to the amount of water-soluble substances, mainly saccharides and simple sugars such as glucose and fructose, as well as dyes, organic acids, and tannins. It serves as a key indicator of the product’s quality and sensory characteristics [24]. The extract content of the beverages ranged from 10.1 to 11.4, indicating that the analyzed herbal shots possess adequate quality in terms of total extract content. Notably, the extract content suggests that the beverages provide a balanced profile of soluble bioactive ingredients, even after infusion dilution. This stability may be advantageous for product development, ensuring consistent sensory characteristics while enabling functional enrichment via herbal components.

Acidity and pH are important physicochemical parameters that determine the taste of the beverage and, in part, its stability during storage, as they influence the thermal treatment of the product. The control sample had the highest total acidity and the lowest pH (1.38 g and 3.03 g malic acid/100 mL, respectively), which can be attributed to the characteristics of the fruits used to create the base. Pear juice is known to contain moderate levels of organic acids, predominantly malic acid, whereas flowering quince is characterized by considerably higher acidity and a stronger flavor profile [25]. The addition of herbal infusions to the base significantly reduced both the acidity and the pH of the drink, likely due to dilution and the lower concentration of organic acids in aqueous extracts. Ultimately, however, the pH remained around 3.0 in all products, ensuring microbiological stability during storage after pasteurization. Comparable reductions have been reported in other functional beverages formulated with herbal materials or water-soluble extracts, where infusion incorporation moderates acidity while maintaining acceptable sensory properties [26]. Despite these modifications, the pH in all formulations remained below 4.0, a threshold commonly regarded as favorable for microbiological stability in pasteurized fruit beverages. Many fruit and herbal drinks with similar acidity profiles demonstrate extended shelf-life without the need for intensive heat treatment or chemical preservatives, as acidic conditions effectively restrict the growth of spoilage microorganisms [27].

Based on the study, it can be concluded that pear–flowering quince juice is the main source of vitamin C in the analyzed shots (96 mg/100 mL). According to Turkiewicz et al. [28], flowering quince is a good source of this compound. Only in shot 1 was there no decrease in vitamin C content. The fortification of the juice with nettle and mint, lime and hops, and white mulberry and yarrow contributed to a reduction in vitamin C content, which may be due to the dilution of the base and the subsequent reduction of the analyzed compound.

In this work, the energy value and osmolarity of the final products were also analyzed (Table 2). The energy value of the products ranged from 34.44 to 42.78 kcal/100 mL, which is similar to other shot products available on the market (among the commercial brands, these include: Red Bull Energy Shot—25 kcal/60 mL; Triebwerk Energy Shot—27 kcal/60 mL; OSHEE Vitamin Shot COMPLEX—10 kcal/100 mL; SuperShot Odporność Purella Superfoods—55 kcal/100 mL). The base had the highest caloric content. The addition of herbal infusions to the pure juice significantly reduced the calorie content. The least caloric were shots 3 and 4, with values ranging from 34.44 to 34.99 kcal/100 mL, respectively. Osmolarity helps determine a beverage’s ability to replenish electrolytes, which is crucial in preventing dehydration. The addition of herbal infusions significantly lowered the osmolarity by introducing additional water into the base. The control sample showed the highest osmolarity (785.5 mOsm/kg), which could promote dehydration. In contrast, sample 2 had the lowest osmolarity (366 mOsm/kg), though it is still classified as hypertonic. Based on the study, it can be concluded that the obtained products belong to the hypertonic category. Based on their physicochemical properties, these products may have the potential to support nutritional replenishment after physical exercise or during periods of increased physiological demand; however, such effects would need to be verified in further studies, preferably at the in vivo level.

The total sugar and sugar alcohols content in the booster beverages ranged from 9.79 to 11.68 g/100 mL (Table 3). The highest total sugar content was recorded in the pear–flowering quince juice, and generally, the starting base (pure juice) determines the qualitative and quantitative sugar content in all prepared formulations. According to a study by Marat et al. [18], flowering quince is low in simple sugars, so pear was the main source of these sugars. Depending on the variety, pear fruits can contain between 5.56 and 10.46 g of total sugars per 100 g [29]. In all beverages, fructose was the dominant sugar (ranging from 6.8 to 5.73 g/100 mL), while glucose (1.7 to 1.45 g/100 mL), sorbitol (2.32 to 1.97 g/100 mL), and sucrose (0.86 to 0.49 g/100 mL) were present in smaller amounts. The addition of herbal infusions to the base resulted in a significant reduction in total sugar content, although it did not notably affect the levels of fructose, sorbitol, or glucose in the individual samples. The inclusion of herbal infusions in shots 1 and 2 had a particularly strong impact on reducing sucrose content, which is considered a beneficial outcome. In addition, the obtained products feature an interesting sugar profile, as, apart from fructose, which is typical for fruits, they contain sorbitol (approximately 2%) and, in lower concentrations, glucose (1.5%) and sucrose (<1.0%). Sorbitol is known to be non-cariogenic and to have a lower glycemic impact than sucrose [30]; however, at the concentration observed in this study its functional relevance for health outcomes should be interpreted with caution. While sorbitol may contribute to the qualitative characteristics of the beverages by partially replacing sucrose, further research would be necessary to determine whether such levels can influence dental health, glycemic response, or other metabolic effects in vivo.

The mineral content (Ca, Na, K, Mg, Fe, Zn) is presented in Table 4. Shot 2, which contained added mint and nettle, had the highest total mineral content (26 mg/100 mL). In contrast, shot 0 had the lowest mineral content (12 mg/100 mL). The enrichment of the base with herbal mixtures led to a significant increase in calcium content across all analyzed samples. The highest concentration of calcium was found in shot 2 (8 mg/100 mL), resulting from the addition of mint and nettle, both of which are recognized as rich sources of this mineral. In contrast, the lowest calcium content was found in shot 3 (5 mg/100 mL). Calcium plays several essential roles in the human body, the most important of which include its involvement in the structure of bones and teeth, blood clotting, muscle contraction, enzyme activation, and immune response. Over 99% of the body’s calcium is stored in bones and teeth, where it provides strength and rigidity to the skeletal system [31]. However, 100 mL of the shot with the highest calcium content provides only ~1.00% of the recommended daily intake of calcium, which is set at 1000 mg. Therefore, booster beverages can be considered a supplement to the daily calcium intake, but not a primary source.

The addition of herbal infusions did not significantly affect the sodium concentration in any of the analyzed shots. Sodium content in the samples ranged from 0.7 to 0.9 mg/100 mL, which is considerably lower than the recommended value. According to Urdampilletai et al. [32], to ensure the correct concentration of sodium ions (Na^+^), the sodium content in beverages should range from 0.5 to 1.5 g/L. Despite their low sodium content, the shots may serve as a beneficial dietary alternative for the prevention of cardiovascular disease, especially for people who need to limit sodium intake in their daily diet. Potassium content ranged from 6 to 8 mg/100 mL. A significant decrease in potassium content was observed in shot 2 (6 mg/100 mL) compared to shot 0 (7 mg/100 mL). The addition of herbal infusions did not significantly affect potassium content in shots 1, 3, and 4. Potassium deficiency is more commonly observed in people with hypertension. Therefore, it can be concluded that the final products—excluding shot 2—may be beneficial dietary supplements to support normal cardiovascular and muscular function; however, confirmation of this requires further in vivo research.

It was observed that fortification of the juice with herbal infusions increased the magnesium content in all samples except shot 1. Notably, shots 2, 3, and 4 showed a significant increase in magnesium levels compared to shots 0 and 1. The highest magnesium content was recorded in shot 2 (10 mg/100 mL), which contained mint and nettle, followed by shot 3, enriched with small-leaved lime and hops (8 mg/100 mL), and shot 4 with white mulberry and common yarrow. In contrast, the lowest magnesium content was found in shot 1 and the pure juice (0.3 and 0.4 mg/100 mL, respectively). Low serum magnesium is independently associated with an increased risk of type 2 diabetes [33]. Magnesium is also a crucial component of bone tissue and plays an important role in nervous system function and muscle activity. The analyzed herbal beverages, especially shot 2, may serve as a valuable source of this macronutrient in the daily diet.

It is worth noting the significant increase in iron content in shot 4 (5 mg/100 g), likely due to the inclusion of white mulberry and common yarrow. In contrast, the addition of herbal infusions to shots 0, 1, 2, and 3 did not result in significant changes in the content of this element. The high iron content in shot 4 makes it particularly suitable as a dietary supplement for the prevention and support of anemia treatment [34].

Trace amounts of zinc were detected in shots 1, 3, and 4 (ranging from 0.01 to 0.03 mg/100 mL). Zinc plays a crucial role in antioxidant defense mechanisms, particularly in people with type 2 diabetes. Additionally, it aids in reducing and neutralizing free radicals, helping to protect cells from oxidative stress [35]. Although the zinc content in the analyzed shots is low, they still offer a useful source of this important micronutrient, which is frequently lacking in the average diet.

### 2.2. Bioactive Compound Contents in the Functional Beverages

The polyphenolic content—comprising four subclasses: phenolic acids, flavonols, monomeric and dimeric flavan-3-ols, and polymeric procyanidins—is presented in Table 5. The highest total polyphenol content was found in shot 2 (579 mg/100 mL). Thus, shot 2, containing mint and nettle, was identified as the richest source of polyphenols among the fortified beverages, with the exception of polymeric procyanidins, which were more abundant in other formulations. In contrast, the lowest polyphenol content was recorded in shot 0 (495 mg/100 mL). This indicates that all herbal additives contributed to enriching the juice with polyphenolic compounds—an extremely important trend in the development of these formulations.

The addition of herbal infusions contributed to an increase in phenolic acid content in all samples, with the exception of shot 3, which exhibited the lowest concentration of these compounds. Mint and nettle (added to shot 2) are known to be rich in phenolic acids such as caffeic acid, p-coumaric acid, 5-*O*-caffeoylquinic acid, and others [8,36]. As a result, the inclusion of these herbs led to a significant increase in phenolic acid content, reaching 17 mg/100 mL (representing over a 100% increase in this fraction of polyphenolic compounds). The addition of a mixture of lemon balm and horsetail, and white mulberry and common yarrow, also enriched the final beverages with phenolic acids by 80% and 42%, respectively. Many herbs, such as horsetail, lemon balm, nettle, mint, yarrow, and mulberry, contain valuable phenolic acids with strong antioxidant and anti-inflammatory effects. Compounds like caffeic, ferulic, rosmarinic, and gallic acids support the body’s natural defense mechanisms and protect cells from oxidative stress. Lemon balm and mint are rich in rosmarinic acid, while nettle and mulberry have a beneficial effect on metabolism and blood sugar management due to their high concentrations of caffeic and chlorogenic acids. In turn, yarrow and horsetail contain caffeic and ferulic acids, which contribute to the anti-inflammatory properties of these plants [37,38].

In addition, a significant increase in flavonol content was observed following enrichment with herbal infusions. The highest flavonol content was found in shot 2 (25 mg/100 mL), which was enriched with mint and nettle. According to Durovic et al. [36], nettle is rich in flavonols such as quercetin, rutin, and kaempferol. Similarly, Pavlesić et al. [8] reported that mint contains high levels of quercitrin, rutin, naringin, and chrysin. These findings explain the increase in the concentration of this fraction in shot 2. The remaining products enriched with herbal infusions also showed a significantly higher content of flavonols compared to the control juice. In the product with the addition of lemon balm and horsetail, the flavonol content was 13 mg/100 mL, while sample 3, enriched with small-leaved lime and hops, contained 7 mg/100 mL. Beverages with white mulberry and common yarrow had 10.66 mg flavonols per 100 mL. The herbal additives used are rich sources of flavonols, which accounts for the increase in this compound group across all formulations. This is because herbs and edible flowers are considered some of the richest sources of this fraction of polyphenolic compounds, with concentrations several times higher than those found in fruits or vegetables on a dry weight basis. This is mainly due to the fact that the leaves and flowers of plants, which are most exposed to environmental factors such as light, accumulate flavonols, which play a protective role in the plants [39,40].

Polymeric proanthocyanidins were the dominant fraction of polyphenolic compounds in all final products, including the control sample. They accounted for 64% (product 2) to 81% (control), with the highest concentration observed in the control juice (402 mg/100 mL) and in products 3 and 4 (410 and 404 mg/100 mL, respectively). Although other authors indicate a high concentration of polymerized compounds in herbs, the addition of infusions in the obtained products did not show an increase or modulation of these compounds. However, a significant increase in the concentration of monomeric and dimeric flavan-3-ols was observed, especially in the product with the lowest concentration of polymerized forms (product 2). This is noteworthy, as literature data directly indicate that herbs generally contain significantly fewer monomeric and dimeric forms than polymerized ones [41,42,43]. This phenomenon may be attributed to the decomposition of procyanidin polymers into their monomeric and dimeric forms. The high temperature used in preparing the infusions may contribute to the degradation of polymers into smaller forms, including catechins, epicatechins, or procyanidins B1 and B2. Another factor influencing this process could be the acidic environment and the presence of antioxidants. It is also worth noting that interactions between polyphenolic compounds from different fractions often lead to an equilibrium between oligomers and monomers of procyanidins [44,45,46]. Thus, several of the aforementioned components influenced the final polyphenolic profile of the obtained formulations.

Based on the analysis of total polyphenol content, it can be concluded that shot 2, which contained mint and nettle, provides the highest amount of polyphenols. The significant differences in polyphenol content are likely attributed to the type of herbal infusions used. In particular, the mint–nettle formulation showed the highest content of flavonols, phenolic acids, and monomeric flavan-3-ols, which are polyphenol classes known for their ability to modulate antioxidant processes. According to epidemiological research, long-term consumption of plant polyphenol-rich diets, especially those containing phenolic acids and flavonols, may help protect against inflammation, viral infections, cancer, cardiovascular diseases, diabetes, osteoporosis, and neurological disorders [47,48]. However, these potential health implications cannot be directly inferred from the present study and would require additional in vivo confirmation.

### 2.3. Bioaccessibility and Bioavailability of Polyphenolic Compounds in the Functional Beverages

In addition to analyzing individual polyphenolic compound fractions in the final products, a bioaccessibility and bioavailability assessment of polyphenolic compounds was performed (Table 5) to determine whether the addition of herbal infusions modulates their bioaccessibility and/or bioavailability. Following a three-stage simulated digestion process (oral cavity → stomach → small intestine), phenolic acids and flavonols exhibited the highest bioaccessibility, with the amount of compounds potentially available after the small intestine stage largely influenced by the formulation composition. The highest bioaccessibility of flavonols and phenolic acids was observed in product 1 (33% and 14% of the initial content, respectively) and product 2 (26% and 18%, respectively). While the lemon balm–horsetail and mint–nettle mixtures were the most effective sources of these compounds, the specific phenolic profiles of the infusions may also have contributed significantly to the observed bioaccessibility. Plants from the *Lamiaceae* family, such as mint and lemon balm, are particularly rich in rosmarinic acid [49,50]. This compound is relatively stable under digestive conditions due to its polarity, which enhances water solubility and facilitates release from the food matrix, especially in liquid formulations where fiber and protein content is limited. Moreover, rosmarinic acid remains stable in the acidic environment of the stomach, resulting in minimal loss at this stage. Consequently, its bioaccessibility in the small intestine can reach up to 35% in selected herbal extracts [51]. The highest bioaccessibility of flavonols in products containing these herbal blends likely stems from their high content of quercetin and luteoin glycosides, which are considered highly water-soluble and readily liberated from plant matrices [52,53]. They also exhibit high tolerance to changing pH, which may be crucial during digestion. Furthermore, other researchers have demonstrated the synergistic effects of rosmarinic acid and flavonols. Rosmarinic acid has been shown to stabilize the polyphenolic matrix, which may improve flavonol bioaccessibility. This stabilization may result from several mechanisms of action. Specifically, polyphenol-polyphenol complexes may form through weak hydrogen bonds or π–π starching, protecting the flavonols from degradation at earlier stages. Furthermore, rosmarinic acid, due to its high polarity, may act as a carrier for less polar compounds, such as quercetin, or as a powerful antioxidant, protecting them from oxidation [51,54,55].

The next groups of compounds analyzed for bioaccessibility were polymeric procyanidins, and flavan-3-ols in both monomeric and dimeric forms. While the bioaccessibility of procyanidin polymers remained approximately 7% across all products, significant variability was observed for the dimeric and monomeric forms of flavan-3-ols, depending on the product type and the herbal blend added to the beverage. The highest bioaccessibility of flavan-3-ols was observed in the base (pear–flowering quince juice) and product 2 with added mint and nettle, at 11.87% and 10.38% of the available pool of compounds, respectively. The remaining products had a bioaccessibility of flavan-3-ols of approximately 6%. The high bioaccessibility of the 100% juice likely results from its simple matrix, which is not additionally loaded with high molecular weight compounds that could bind flavan-3-ols. The high bioaccessibility in product 2 is again due to the presence of rosmarinic acid [55], which exhibits high antioxidant capacity and increases the solubility of compounds. Menthol may also be an additional supporting factor, as it acts as a surfactant, facilitating the dispersion of polyphenols and limiting their precipitation [56]. Therefore, although product 1 contains a lot of rosmarinic acid (from lemon balm), due to the lack of menthol, bioaccessibility is much lower (apart from the starting effect, i.e., many more flavan-3-ols were determined in product 2.

Analysis of the bioavailability of polyphenolic compounds in the tested products showed that their levels depend not only on the initial content and profile of individual compounds, but also on their bioaccessibility. The highest total bioavailability relative to the available pool of compounds after digestion (11.16% of total polyphenols) was observed in product 2 (mint + nettle). A relatively high bioavailability was also recorded for product 1 (lemon balm + horsetail)—7.83% of the post-digestion pool. The high bioavailability in these two cases can be attributed to the synergy of several factors: (i) the presence of quercetin and/or luteolin glycosides, which enhance flavonoid solubility; (ii) the high content of rosmarinic acid (in lemon balm and mint), which stabilizes flavan-3-ols due to its strong antioxidant activity; (iii) the relatively simple composition of the tested matrices, where the absence of fiber or high protein content limits the formation of polyphenol–protein or polyphenol–fiber complexes.

In general, however, it should be emphasized that the bioavailability of the total polyphenolic compounds in the small intestine was relatively low compared to other reports in the literature. For example, Baeza et al. investigated widely consumed beverages (chamomile tea, yerba mate and a green/roasted coffee blend) and reported polyphenol recovery after in vitro gastrointestinal digestion of about 57% for yerba mate and 78% for the coffee blend, indicating relatively high stability of the phenolic fraction in these matrices [57]. In contrast, cocoa bean shell–based beverages showed total polyphenol bioaccessibility of approximately 50–55%; within this group, individual flavan-3-ols such as epicatechin were poorly bioaccessible (~10–13%), while catechin and some procyanidins could even exceed 100% bioaccessibility due to depolymerization and release from the matrix [58]. *Hibiscus sabdariffa* beverages exhibited a marked reduction in the number of identifiable bioactive compounds after digestion (from 35 to a maximum of 15), with phenolic acids being the least bioaccessible group, while flavonoids were more widely retained in the bioaccessible fraction [59]. Similarly, in *Aloe vera* juices, some phenolics showed moderate to high bioaccessibility in the intestinal phase (e.g., ~49–55% for hesperidin and epicatechin), whereas others were strongly degraded (<30% bioaccessibility), again highlighting the selective stability of flavonoids vs. phenolic acids in liquid systems [60]. In the products we describe, the relatively low bioavailability is most likely due to the fact that the initial matrix contains a very high proportion of polymeric procyanidins (approximately 80%), which are not absorbed in the small intestine. Their limited absorption is due to the high molecular weight of these compounds and the absence of specific transporters in the intestinal epithelium for such large molecules, and their physicochemical properties (large size and hydrophilicity) prevent passive diffusion across the enterocyte membrane [61]. Further research is needed in this area, as numerous literature data indicate that polymeric procyanidins, although not absorbed into the bloodstream in the small intestine, pass to the large intestine, where they are degraded by the intestinal microbiota into highly biologically active low-molecular-weight phenolic metabolites [61,62]. Therefore, their significant potential should continue to be considered, including in the products discussed here. A markedly better effect at the small intestine stage was observed for phenolic acids, flavonols, and flavan-3-ols, highlighting the significant potential of food fortification with herbal blends, particularly those that introduce new bioactive compounds into the product. This approach promotes synergy in both bioaccessibility and bioavailability, while also broadening the overall health-promoting effects.

### 2.4. Analysis of Health-Promoting Potential of the Obtained Products Using In Vitro Methods

#### 2.4.1. Antioxidant Activity of Analyzed Functional Beverages

**Figure 1:**
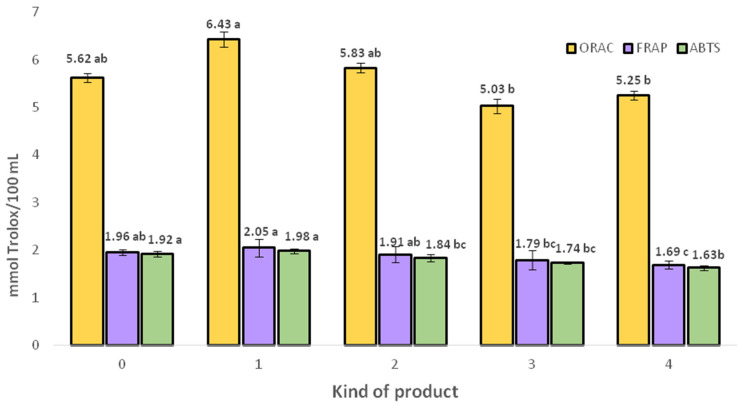
Antioxidant activity of obtained functional beverages. Data are given as mean ± SD (*n* = 3). Mean values within columns of the same color with different letters (a–c) are significantly different (homogenous groups) at *p* ≤ 0.05; full product characteristics in Table 1.

The antioxidant activity (ORAC, FRAP, ABTS) is presented in Figure 1. The addition of herbs preserved antioxidant activity in the final products. The ORAC method demonstrated the greatest increase in antioxidant activity, ranging from 5.03 to 6.43 mmol Trolox/100 mL. Mixtures such as lemon balm–horsetail and mint–nettle enriched the final beverages with antioxidant properties by 14% and 4%, respectively.

The FRAP method also showed that the addition of herbal infusions supports antioxidant efficacy, with the exception of shot 4, which exhibited the lowest antioxidant effect. The increases observed with the FRAP method were smaller than those seen with ORAC, ranging from 1.69 to 2.05 mmol Trolox/100 mL.

Similarly, the ABTS assay showed smaller values, ranging from 1.63 to 1.98 mmol Trolox/100 mL. All herbal mixtures supported antioxidant effects, except shot 4, which consistently exhibited the lowest antioxidant activity. Considering all the methods applied, shot 4 was the least effective in terms of antioxidant activity, despite various authors reporting its antioxidant properties.

Polyphenols play an important role in exhibiting strong antioxidant capacity. According to Gil et al. [63] and Nowicka et al. [64] the polyphenol content, especially flavan-3-ols and phenolic acids, is strongly correlated with antioxidant activity. However, in the developed formulations, in addition to a moderate positive correlation between the content of phenolic acids and antioxidant activity (*R* = 0.690 phenolic acid/ORAC), a moderate relationship was also observed for flavonols (*R* = 0.573, flavonols/ORAC). In this context, it can be concluded that the boosted beverages have the potential to serve as products for the prevention of chronic diseases, such as neoplastic diseases, cardiovascular diseases, diabetes, and neurodegenerative diseases. The increase in antioxidant activity observed in the herbal-fortified beverages is consistent with the results of Owczarek et al. [65], who demonstrated that enriching fruit juices with herbal infusions significantly increases total phenolic content and antioxidant potential. Similarly, Fu et al. [66] showed that herbal and tea infusions are rich in phenolic compounds and exhibit strong antioxidant properties, with phenolic acids and flavonoids playing a key role in modulating this activity. The relationship between polyphenolic content and antioxidant behavior is also supported by Chandrasekara and Shahidi [67], who indicate that herbal beverages can act as donors of bioactive compounds and may contribute to the prevention of chronic diseases. This is consistent with the results obtained for shots 1 and 2, confirming that herbal fortification is an effective strategy for improving the antioxidant potential of fruit-based beverages.

#### 2.4.2. Ability to Inhibit α-Amylase, α-Glucosidase, Pancreatic Lipase, and Lipoxygenase-15 of Functional Beverages

The inhibition effects of α-amylase, α-glucosidase, pancreatic lipase, and lipoxygenase-15 are presented in Table 6.

Inhibiting pancreatic lipase, α-amylase, and α-glucosidase can slow the digestive process and, consequently, reduces glucose absorption in the small intestine through enterocytes, which subsequently results in smaller blood glucose peaks. Effective inhibitors of these enzymes are considered glucose-lowering and fat-reducing agents. A slow and prolonged release of glucose into the bloodstream is important in managing hyperglycemia and type 2 diabetes. Furthermore, inhibiting these digestive enzymes may help achieve satiety and contribute to weight loss [68].

The inhibition of α-amylase, expressed as IC_50_, ranged from 0.366 to 0.572 mg/mL. The strongest inhibition was found in shot 2, which was enriched with nettle and mint (0.366 mg/mL), while the weakest inhibition was found in shot 4, containing white mulberry and yarrow (0.572 mg/mL). According to Al-Mijalli et al. [69], peppermint essential oil shows significant inhibition of α-amylase. Additionally, Altamimi et al. [70] reported that nettle leaves possess a high α-amylase inhibition potential. Therefore, the α-amylase inhibition values presented here support the potential antidiabetic properties of the beverages tested.

The inhibition of α-glucosidase, expressed as IC_50_, ranged from 0.02 to 1.523. The strongest inhibition was observed in shot 0 (pure juice). According to Wang et al. [19] and Mohebbi et al. [71], pear and flowering quince fruits are known for their strong inhibition capabilities. The weakest inhibition was found in shot 2, with mint and nettle, and shot 4, with common yarrow and white mulberry (1.523 and 1.495 mg/mL, respectively), despite the good inhibition values for these herbs against α-glucosidase described by others [12,69,70,71]. The inhibitory activity of the herbal formulations was several dozen times weaker than that of acarbose—a drug used in the treatment of type 2 diabetes (IC_50_ = 0.02 mg/mL). This confirms that the obtained products have no practical potential as α-glucosidase inhibitors, even though the juice base itself exhibited a very strong inhibitory effect. These findings show that the herbal mixtures did not enhance the inhibition capabilities.

Differences in inhibition may be influenced by the composition of polyphenols in herbal mixtures. Although several authors have reported significant antidiabetic activity in herbs, the addition of herbal infusions to the obtained products did not demonstrate an enhancement of the inhibition effect against α-glucosidase. However, it did help to increase the inhibitory effectiveness toward α-amylase. Usually, stronger inhibition is observed for α-glucosidase in plant materials and products based on them, as compared to α-amylase. The observed trend may be due to differences in the composition of plant extracts and the enzyme structure of the enzymes, which could cause some plant products to inhibit amylase more effectively. In addition, enzymes and chemical compounds present in herbs, as well as the acidic environment, may also influence this mechanism. According to Nowicka et al. [72], quercetin glucosides attenuate the action of glucose transporters. In this context, the combination of these components played a role in shaping the final profile of the formulations.

In this study, the ability to inhibit pancreatic lipase was also assessed (Table 6). The results showed that the boosted beverages effectively inhibited pancreatic lipase. The inhibition, expressed as the IC_50_, ranged from 0.005 to 0.063 mg/mL. Pure juice demonstrated the highest potential to inhibit lipase, with an IC_50_ of 0.05 mg/mL. Strong inhibition against lipase was also observed in shot 1, enriched with lemon balm and horsetail; shot 3, enriched with hops and small-leaved lime; and shot 4, containing common yarrow and white mulberry, with IC_50_ values of 0.057, 0.056, and 0.057 mg/mL, respectively. Each of these variants is therefore a potent inhibitor of the enzyme. This finding is particularly valuable, as pancreatic lipase inhibitors are important tools in the fight against obesity by reducing the caloric value of the diet through the inhibition of fat digestion. However, it should be noted that this effect may also decrease the absorption of other bioactive compounds, including fat-soluble vitamins and additional hydrophobic constituents. Therefore, although the developed formulations demonstrated promising inhibitory activity against both α-amylase and pancreatic lipase, further research is required to assess potential undesirable consequences associated with reduced nutrient absorption.

Fortification of juices with herbal mixtures can also modulate the anti-inflammatory properties of the final formulations. In this work, the ability of the developed products to inhibit LOX-15 was assessed. LOX-15 is one of the most important enzymes in the metabolism of unsaturated fatty acids. Dysregulated expression of this enzyme is closely associated with the pathology of various cancers and tumor progression, making it important to identify effective inhibitors of this enzyme. The study demonstrated high inhibitory potential against LOX-15, with IC_50_ values ranging from 0.111 to 0.295 mg/mL. Shot 4, enriched with common yarrow and white mulberry, showed the strongest inhibition of LOX-15 (0.111 mg/mL), while the weakest inhibition was observed in shot 2 (mint and nettle). This study also showed a strong positive correlation between the content of polymeric procyanidins and anti-inflammatory activity (*R* = 0.775), classified as strong according to Dancey and Reidy [73]. Other authors have reported a similar trend, indicating that procyanidin polymers can regulate the NF-κB pathway, which plays a key role in the inflammatory response. They also modulate anti-inflammatory activity by reducing oxidative stress [74]. Additionally, the authors note that the effectiveness of anti-inflammatory action is influenced by the degree of polymerization, with dimers and trimers being much weaker inhibitors of inflammation than procyanidin polymers [75,76].

### 2.5. Sensory Evaluation of the Obtained Functional Beverages

**Figure 2:**
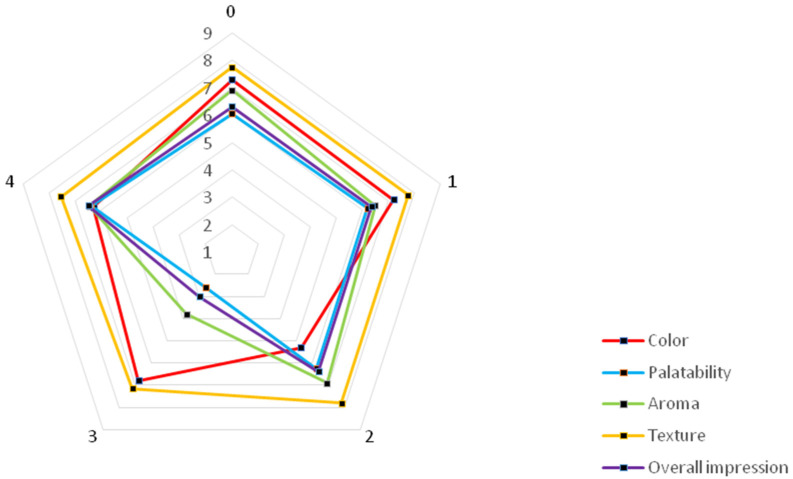
Sensory evaluation of obtained beverages (0–1—individual products as described in Table 1).

To determine consumer market acceptability, a sensory analysis was conducted in which panelists evaluated the color, aroma, palatability, texture, and overall rating of the final beverages (Figure 2).

The control sample, which served as the base for the developed beverages, received the highest acceptability in terms of color (7.3). The addition of a mixture of lemon balm and field horsetail (shot 1) was also well rated (7.2). The least desirable color was observed in shot 2, enriched with mint and nettle (5.3). The addition of these herbs led to a darkening of the sample and the appearance of a red color, according to the CIELab system. The additions of small-leaved lime and hops (shot 3), and white mulberry and yarrow (shot 4), were rated within the “moderately like” and “quite like” range.

The palatability of shots 0, 1, 2, and 4 was rated within the range of “moderately like” to “quite like.” The trait was rated more favorably after the addition of herbs (shots 1, 2, and 4) compared to the control sample. Shot 3 (hops and lime) received the lowest rating for this trait, with a score of 2.6. Evaluators assigned the lowest scores to this variant due to the characteristic bitterness of hops, which resulted in undesirable sensory notes in the finished product. The aroma of shots 0, 1, 2, and 4, as well as palatability, was rated between 6.0 and 6.9. The control sample with pear–flowering quince juice and shot 2 (mint and nettle) were rated the best. The sample with lemon balm and field horsetail (shot 1) was rated worse than shots 0 and 2, but better than shot 4 (white mulberry and yarrow). The addition of hops and small-leaved lime (shot 3) negatively impacted the rating (3.8) due to the characteristic aroma of hop cones.

The consistency of all shots was within a similar range (7.2–7.8). Shot 3 (small-leaved lime, hops) received the lowest rating, likely due to the astringency and lack of smoothness caused by the addition of hops. The addition of yarrow and white mulberry (shot 4) was less acceptable than shots 0, 1, and 2.

In the overall evaluation, the herbal shot with white mulberry and yarrow (shot 4) received the highest rating (6.5). Shot 3, with small-leaved lime and hops, received the lowest rating. It was also noted that shots 1 (lemon balm and field horsetail) and 2 (mint and nettle) scored slightly better compared to the control sample.

### 2.6. PCA

**Figure 3:**
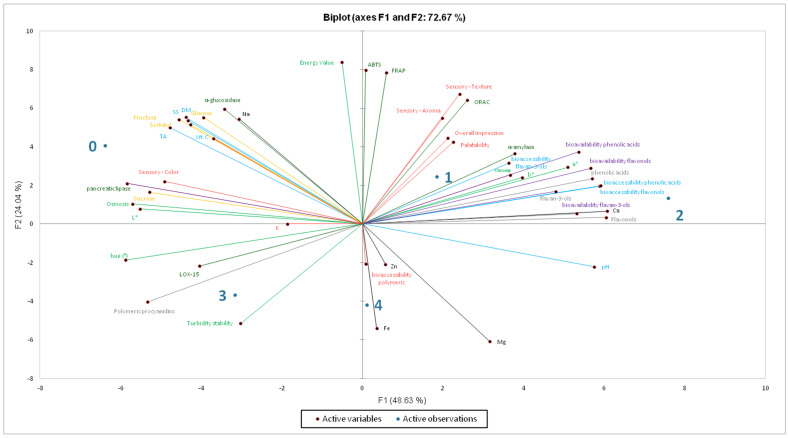
PCA of obtained products (0–1—individual products as described in Table 1).

Figure 3 presents a principal component analysis (PCA) performed for the obtained beverages. The first principal component (F1; 48.63%) reflected a functional gradient, distinguishing products with high antioxidant activity, favorable sensory perception, and high bioavailability of phenolic acids, flavonols, and flavan-3-ols (positive loadings) from formulations characterized by high sugar content, low bioavailability, and reduced antioxidant performance (negative loadings). The second principal component (F2; 24.04%) separated sensory attributes, largely modulated by sugar content, from technological parameters and physicochemical stability associated with polymerized compounds, indicating that matrix composition influenced sensory expression independently of antioxidant behavior. Together, F1 and F2 explained 72.67% of the total variance, confirming strong dimensionality reduction and a meaningful differentiation among product characteristics.

A clear functional gradient was observed along PC1 between bioactive performance and physicochemical characteristics arising from the pear and flowering quince matrix. Products 2 and 1 were positioned furthest on the positive side of PC1 and were strongly associated with antioxidant properties, as well as with the bioaccessibility and bioavailability of phenolic acids, flavonols, and monomeric and dimeric flavan-3-ols, in addition to favorable sensory attributes. This confirms that herbal enrichment based on mint–nettle and lemon balm-horsetail combinations delivered the most pronounced functional and sensory benefits among all formulations. Product 4 was also located on the positive side of F1 but on the negative side of F2. This positioning indicates an association with selected mineral elements and variables related to the bioaccessibility of polymeric proanthocyanidins. Hence, this formulation, although linked with polymerized phenolics and potentially susceptible to technological instability, simultaneously retains high antioxidant potential and favorable sensory appeal. Conversely, sample 3 was located on the negative side of F1 and F2, and was primarily associated with polymeric procyanidins, strong LOX-15 inhibitory activity and reduced turbidity stability. This suggests that its bioactive profile was dominated by high-molecular-weight phenolics with strong structural and colloidal effects but without proportional antioxidant efficacy or sensory enhancement. High polymerization translated into technological differentiation rather than comprehensive functional benefits. Product 0 (100% juice) was located on the negative side of F1 and the positive side of F2, showing strong associations with sugar content and low bioavailability. The juice therefore represents a typical base matrix that can be made functional through enrichment with herbal infusions.

PCA also indicated strong directional clustering between antioxidant assays (ORAC, FRAP, ABTS) and phenolic fractions (phenolic acids, flavonols, and monomeric and dimeric flavan-3-ols). Bioaccessibility and bioavailability vectors were aligned with the same functional axis, confirming that improved release and absorption of low-molecular-weight phenolics enhanced oxidative capacity. In contrast, enzymatic inhibition (α-glucosidase, pancreatic lipase, and LOX-15) formed vectors oriented away from the antioxidant domain, indicating that inhibitory activity is modulated predominantly by high-molecular-weight or polymerized phenolic structures.

Finally, PCA showed that even minimal supplementation with herbal infusions significantly improved the functional quality of pear–flowering quince juice beverages, enhancing antioxidant potential, sensory performance, and the bioavailability of polyphenolic compounds.

## 3. Materials and Methods

### 3.1. Plant Material

The herbs used to prepare all four infusions were obtained from commercial sources. The production utilized lemon balm, horsetail, small-leaved lime, nettle, mint, and hops from the producer “Dary Natury” (Grodzisk, Poland), white mulberry from the company “Flos” (Bovezzo, Italy), and yarrow herb from the company “Herbapol” (Lublin, Poland).

The pear fruit (“Faworytka” cv.) and the flowering quince (“Cameo” cv.) used for juice production were collected at full technological maturity stage from the Central Research Centre for Cultivar Research in Zybiszów near Wrocław (51°04′ N, 16°78′ E) in the second half of August 2023 and from the Experimental Orchard in Wrocław (51°06′ N, 17°02′ E) in October 2023, respectively. Immediately after harvesting, the whole fruits were processed. The pomace fruits underwent washing, homogenization, and enzymatic treatment (*t* = 30 min; *T* = 36 °C; Pectinex concentration = 0.05%). The fruits were then squeezed using a hydraulic press (30-ton shop press, “Junior”) to obtain raw juices.

### 3.2. Herbal Shots Preparation

The production process for the shots began with the preparation of an infusion made from selected herbs and pear–flowering quince juice. To do this, a mixture of two herbs in a 1:1 (*w*/*w*) ratio was prepared, with 17.5 g of the mixture weighed and infused with 140 g of water (*T* = 80–85 °C). The infusion was brewed (*t* = 20 min) and then gravity-filtered using qualitative paper filters to obtain 90 g of pure extract. The following herb combinations were used to prepare the infusion: lemon balm with horsetail (*M. officinalis* and *E. arvense*)—product 1, mint with nettle (*Mentha* × *piperita* and *U. dioica*)—product 2, small-leaved lime with hops (*T. cordata* and *H. lupulus*)—product 3, and white mulberry with common yarrow (*M. alba* and *A. millefolium*)—product 4. The herbal blends were selected based on their sensory properties and compatibility with the juice matrix. In addition, their documented health-promoting potential, particularly antioxidant and anti-inflammatory effects, was considered. The blends included botanicals rich in complementary phytochemical classes (e.g., rosmarinic acid, quercetin glycosides, menthol), which were intended to enhance both functional performance and sensory acceptability. All herbs are traditionally used in food applications and were commercially available, ensuring safety, practicality and reproducibility of the formulations.

The juice base was obtained by mixing the pear juice and flowering quince juice in a 4:1 (*w*/*w*) ratio. The next step involved mixing the infusion with the pear–flowering quince juice to achieve a final product containing 15% infusion. The mixture was then heated in a Thermomix device (*t* = 10 min; *T* = 98 °C). The product was poured into glass jars of approximately 70 mL capacity, tightly sealed, and subjected to self-pasteurization (*t* = 10 min; *T* = 98 °C). Finally, the ready shots were cooled under running water (*t* = 10 min), after which the product was ready for storage and further use.

**Figure 4:**
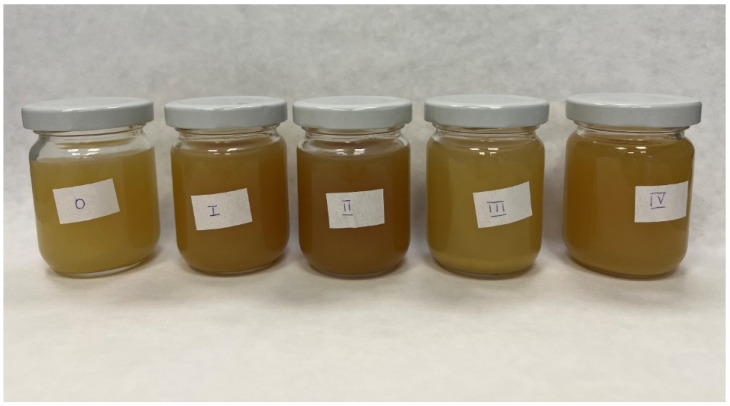
Prepared beverages (from left to right): 0—control samples, I (1)—product 1 with lemon balm and horsetail; II (2)—product 2 with mint and nettle; III (3)—product 3 with small leaves lime and hops; IV (4)—product 4 with white mulberry and common yarrow).

Selected herbal blends were developed to complement the fruit juice base (pear–flowering quince juice) and enhance the overall health-promoting properties of the beverages, including enhanced bioavailability due to the distinct profiles of each blend. Each blend incorporates herbs with well-documented antioxidant, anti-inflammatory, and bioactive properties, aiming to achieve a synergistic effect. Lemon balm and horsetail support antioxidant activity and contribute essential minerals [5,6], while mint and nettle aid digestion and exhibit anti-inflammatory potential [7,8]. The combination of small-leaved lime and hops provides antioxidant and chemopreventive benefits [9,10], while white mulberry and yarrow support glycemic regulation and display additional anti-inflammatory effects [11,12]. Overall, the 15% herbal infusion in each formulation was optimized to enrich the beverage’s bioactive content without compromising taste, resulting in the creation of the functional beverages presented in Table 1 and in Figure 4.

### 3.3. Physical Parameters

Color parameters were measured under reflected light using a 10° standard observer and D65 illuminant with a ColorQuest XE instrument (HunterLab, Reston, VA, USA), following the guidelines provided in its user manual. The test solutions were placed in 2 cm cells. The color parameters L*, a*, and b* were then recorded for the analyzed samples, where L* represents lightness, a* indicates red for positive values and green for negative values, and b* indicates yellow for positive values and blue for negative values. Chroma (C*) and hue angle (h°), which provide a more complete interpretation of color characteristics.

Chroma (C*) was calculated as: $C^{*}\;=\;\sqrt{a^{*2}+b^{*2}}$, and represents the saturation or intensity of the color.

The hue angle (h°) was derived according to the following equation: $h{}^{\circ}=\arctan\left(\frac{b^{*}}{a^{*}}\right)$, and expressed in degrees (0–360°), after appropriate quadrant correction based on the signs of a* and b*. The hue angle represents the perceived dominant color tone.

The turbidity stability of the herbal shots was measured using a Turbiquant 3000 IR instrument from Merck (Darmstadt, Germany), following the guidelines provided in its user manual. The results are expressed as %NTU. All measurements were performed in triplicates and are presented as the average value ± SD.

### 3.4. Calorific Value

The analysis was conducted following the manufacturer’s recommendations for the calorimeter. Energy values were determined using a C 200 calorimeter paired with an RC 2 basic recirculation cooler (IKA, Wilmington, NC, USA).

A cotton thread with known calorific value (IKA, Wilmington, NC, USA) was mounted on the ignition wire of the calorimetric bomb C 5010 (IKA, Wilmington, NC, USA). A 1 mL sample was placed in quartz crucibles. If necessary, auxiliary materials such as combustion bags, parafilm, or paraffin were used to facilitate combustion. Then, the sample was transferred to a calorimetric bomb, and a cotton thread was inserted into it. The calorimetric bomb was sealed and filled with compressed oxygen (35 Ba) using C 248 Oxygen filling station (IKA, Wilmington, NC, USA) to optimize the combustion process. Next, the sample was combusted in the calorimeter and the system measured the temperature increase. The obtained values were converted to 100 mL of sample.

### 3.5. Basic Chemical Composition

Dry matter analysis was conducted following the PN-90-A-75101/03 standard [77]. Extract analysis was performed using a refractometer (ATAGO Pocket PAL-1, Bellevue, WA, USA). Total acidity was measured via potentiometric titration following the PN-EN 12147:2000 standard [78]. The pH value and the amount of NaOH used were recorded using a TitroLine 6000 titrator (SI Analytics, Mainz, Germany). Total acidity was expressed as the amount of malic acid g/100 mL of the product. Active acidity was measured using a pH meter from IQ Scientific Instruments. Osmotic pressure of the shots was measured using the Marcel OS 3000 osmometer (Marcel S.A., Zielonka, Poland), an automated device that enables precise, rapid osmotic analysis based on freezing-point depression. All measurements were performed in triplicates and are presented as the average value ± SD.

### 3.6. Determination of Sugar Content

Chromatographic analysis of sugar content was conducted using a Merck-Hitachi L-7455 liquid chromatograph with an evaporative light scattering detector (ELSD) (Polymer Laboratories PL-ELS 1000, Agilent Technologies, Santa Clara, CA, USA) and a quaternary pump L-7100, equipped with a D-7000 HSM solvent delivery system (Merck-Hitachi, Tokyo, Japan). The chromatographic analysis was conducted using a Carbohydrate ES HPLC Column-W (250 mm × 4.6 mm, 5 µm) (Alltech, Lexington, KY, USA), following the method described by Wojdyło et al. [79]. The content of individual sugars was identified based on standard reference materials. The results are presented as g/100 mL, based on three repetitions.

### 3.7. Determination of Vitamin C

Samples of herbal shots (approximately 10 mL) were transferred into tubes and supplemented with 5, 3, and 5 mL of a 3% metaphosphoric acid (MPA) solution, respectively. The samples were shaken (*t* = 30 min) in a darkroom. After shaking, the mixtures were centrifuged and filtered into vials. The vitamin C content in the herbal shots was determined directly from the centrifuged and filtered samples.

Chromatographic analysis was performed using a Cadenza CX-C18 column (150 × 4.6 mm, 3 μm; Imtakt, Portland, OR, USA), thermostated at 35 °C. The mobile phase consisted of a 15 mM H_3_PO_4_ solution, with isocratic elution at a rate of 1 mL/min. The injection volume was 10 μL, and methanol (MeOH) was used for needle rinsing. Readings were taken at a wavelength of 250 nm. Vitamin C was identified based on standard reference materials.

### 3.8. Determination of Mineral Content by Atomic AAS

The mineralization process was conducted in a sealed reactor placed in a microwave system, a Microwave Digestion System Multiwave GO (Anton Paar, Graz, Austria), according to guidelines provided in its user manual. Mineral content analysis was performed using atomic absorption spectrometry (AAS) with an AA-7000 spectrometer (Shimadzu, Koyoto, Japan) and a hydrogen vapor generator HVG-1 (Shimadzu, Koyoto, Japan) according to guidelines provided in its user manual. The results were expressed as milligrams of element per 100 mL of the sample, based on three repetitions.

### 3.9. Identification and Quantification of Polyphenolic Compounds, Including Polymers Procyanidins

The methods for the identification and quantitative analysis of polyphenols (ultra performance liquid chromatography with photodiode array and fluorescence detectors (UPLC-PDA-FL_) were performed as previously described by Wojdyło et al. [80]. The phenolic compounds were monitored at 280 nm (dihydrochalcones and flavan-3-ols), 320 nm (phenolic acids), 360 nm (flavonols), and 520 nm (anthocyanins). In addition, the analysis of polymeric procyanidins was carried out using the phloroglucinol method, as described by Kennedy and Jones [81]. The results were expressed as milligrams per 100 mL, based on three repetitions.

### 3.10. Analysis of Bioaccessibility and Bioavailability

Simulated gastrointestinal digestion was conducted following the protocol described by Świeca et al. [82] with minor modifications. The experiment was performed on both 100% pear–flowering quince juice and the formulated products. For all samples, approximately 20 g was weighed and mixed with 10 mL of simulated salivary fluid, then thoroughly shaken for 3 min at 37 °C. The pH was adjusted to 3 using 5 mol/L HCl. Next, 30 mL of simulated gastric fluid was added, and the sample was incubated for 200 min at 37 °C. The pH was then adjusted to 6 using 0.1 mol/L NaOH to simulate gastric conditions. Subsequently, 60 mL of a mixture containing bile extract and pancreatic enzymes was added, and each sample was adjusted to pH 7 using 1 mol/L NaOH. Finally, 5 mL of 120 mmol/L NaCl and 5 mL of 5 mmol/L KCl were added to each sample, and the samples were incubated in the dark for 120 min at 37 °C.

Dialysis tubes (Pur-A-Lyzer Maxi 12,000, Sigma-Aldrich, St. Louis, MO, USA) were used to determine bioavailability. Three milliliters of digest were placed into the dialysis tube, which was subsequently immersed in 18 mL of PBS solution and incubated at 37 °C for 120 min. After incubation, individual fractions (digested and post-dialysis) were collected, and their polyphenolic compound content was analyzed according to the protocols described above.

### 3.11. Analysis of Health-Promoting Potential Using In Vitro Methods

#### 3.11.1. Antioxidant Activity

The ABTS^•+^ antioxidant activity assay, as described by Re et al. [83], is based on the reduction in the blue-green color of ABTS^•+^ by antioxidant substances. The decrease in color intensity directly correlates with the concentration of antioxidants in the solution. Absorbance measurements were taken after 6 min at a wavelength of λ = 734 nm using a UV-Vis 2401 spectrophotometer (Shimadzu, Kyoto, Japan).

The Ferric Reducing Ability of Plasma (FRAP) assay, as described by Benzie and Strain [84], is based on the ability of antioxidant substances to reduce Fe^3+^, complexed with tripyridyltriazine (TPTZ), to Fe^2+^, causing a color change from colorless to blue. Absorbance was recorded after 10 min at a wavelength of *λ* = 593 nm using a UV 2401 PC spectrophotometer (Shimadzu, Japan).

The Oxygen Radical Absorbance Capacity (ORAC) assay, as described by Ou et al. [85], involves the decay of fluorescence in a fluorescent substance (typically fluorescein) due to oxidation by free radicals. Antioxidants inhibit changes in fluorescence, thereby preventing the oxidation of the fluorescent substance. Antioxidant activity was measured using an RF5301 PC spectrofluorometer (Shimadzu, Kyoto, Japan) at an excitation wavelength of 487 nm and an emission wavelength of 528 nm. Plates were incubated at 37 °C, with measurements taken every 5 min after the introduction of the AAPH reagent. The analysis duration varied depending on the rate of fluorescence decay.

Calibration curves were prepared for all analytical methods. For the ABTS and FRAP assays, the Trolox concentration range was 0.1–1.0 mmol, while for the ORAC assay it was 12.5–100 µmol Trolox. The results for all methods are expressed as mmol of Trolox per 100 mL of sample ± SD, based on three repetitions.

#### 3.11.2. Ability to Inhibit α-Amylase and α-Glucosidase

The analyses were conducted as previously described by Nowicka et al. [68]. The method is based on a color reaction in which iodine causes amylopectin to turn purple and amylose to turn blue. Both structures are highly polymerized. The implementation of the α-amylase enzyme to the sample containing starch leads to depolymerization, resulting in the subsequent loss of color. The ability to inhibit α-amylase activity was determined by measuring the absorbance using a UV 2401 PC spectrophotometer (Shimadzu, Kyoto, Japan) at a wavelength of 540 nm. The second method involves measuring the amount of glucose released from p-nitrophenyl-α-D-glucopyranoside. Spectrophotometric measurements to assess the ability to inhibit α-glucosidase were performed using the same spectrophotometer at a wavelength of 405 nm. Acarbose was used as the positive control for both enzymes, with half maximal inhibitory concentration (IC_50_) values of 0.35 and 0.02 mg/mL for α-amylase and α-glucosidase inhibition, respectively. The results are presented as IC_50_ (mg/mL ± SD).

#### 3.11.3. Determination of Anti-Inflammatory Capacity by 15-Lipoxygenase (LOX-15) and Lipase Inhibitory Inhibition

The antidiabetic activity of the herbal shots was evaluated using the method described by Nowicka et al. [72]. Absorbance measurements were performed at a wavelength of 560 nm using a Synergy™ H1 reader (BioTek, Winooski, VT, USA). The results are expressed as IC_50_ ± SD (mg/mL).

### 3.12. Consumer Evaluation of the Herbal Shots

The sensory evaluation was conducted at the Faculty of Biotechnology and Food Sciences of Wrocław University of Environmental and Life Sciences, in accordance with ISO 8589:2009 standards [86]. Analyses of color, aroma, palatability, and overall impression of the products were carried out using a 9-point hedonic scale, where 1 indicated “dislike extremely” and 9 indicated “like very much.” For the evaluation, all products were labeled with codes and served in small, transparent plastic glasses. After tasting each sample, panelists neutralized their mouths. Participants were informed that their participation was entirely voluntary, that they could withdraw from the evaluation at any time, and that all responses would remain anonymous.

In addition, we obtained consent from the Scientific Research Ethics Committee to conduct the sensory tests, as approved by resolution no. N0N00000.0020.1.8.2.2024.

### 3.13. Statistical Analysis

Statistical analysis was performed using Statistica software, version 13.3. To compare the mean parameter values among the products, a one-way analysis of variance (ANOVA) was conducted, followed by the Duncan and Tukey tests (*p* ≤ 0.05). To prepare Principal Component Analysis (PCA), XLSTAT (Addinsoft, New York, NY, USA) was used.

## 4. Conclusions

The study demonstrated that fortifying pear and flowering quince juice with herbal infusions is an effective strategy for improving the nutritional, sensory, and functional quality of shot beverages. Even minimal supplementation (15%) significantly modulated the behavior of polyphenolic compounds, increasing their bioaccessibility and bioavailability and, consequently, enhancing antioxidant activity and health-promoting potential. Among all formulations, shots enriched with mint–nettle (product 2), and lemon balm–horsetail (product 1) were the most promising, combining high total phenolic content, strong intestinal availability of phenolic acids, flavonols and flavan-3-ols, and attractive sensory properties. These effects were most likely associated with synergistic interactions between rosmarinic acid, quercetin-type flavonols and menthol, which collectively improved the solubility, oxidative stability and release of low-molecular-weight polyphenols.

Polymeric procyanidins remained poorly bioaccessible but contributed to LOX-15 inhibition and technological variability, suggesting that their significance may extend beyond intestinal antioxidant activity. PCA confirmed that functional improvement was primarily driven by low-molecular-weight phenolics, whereas polymerized fractions affected stability and enzymatic inhibition (LOX-15, α-glucosidase and pancreatic lipase). Collectively, herbal infusions acted as efficient donors of bioactive compounds that effectively modulated product characteristics and imparted new functional attributes.

## Figures and Tables

**Table 1:** Herbal Shot Compositions.

No.	Final Products	
	Juice	Herbal
0	100% pear ÷ flowering quince juice (4 ÷ 1)	
1	85% pear ÷ flowering quince juice (4 ÷ 1)	15% infusion of lemon balm ÷ horsetail (1 ÷ 1)
2	85% pear ÷ flowering quince juice (4 ÷ 1)	15% infusion of mint ÷ nettle (1 ÷ 1)
3	85% pear ÷ flowering quince juice (4 ÷ 1)	15% infusion small leaved lime ÷ hops (1 ÷ 1)
4	85% pear ÷ flowering quince juice (4 ÷ 1)	15% infusion white mulberry ÷ common yarrow (1 ÷ 1)

**Table 2:** Physico-chemical composition of obtained functional beverages.

Parameter		100% Pear–Flowering Quince Juice (0)	No. of Product *			
			1	2	3	4
Colour	L*	51.62 ± 0.08 a	49.39 ± 0.26 d	45.31 ± 0.03 b	51.62 ± 0.16 a	46.79 ± 0.17 c
	a*	−3.52 ± 0.01 a	−0.93 ± 0.05 c	0.28 ± 0.04 d	−3.4 ± 0.09 a	−2.3 ± 0.03 b
	b*	13.46 ± 0.05 b	18.10 ± 0.11 d	16.08 ± 0.11 c	14.50 ± 0.42 a	14.30 ± 0.27 a
	hue (°)	104.66 ± 0.07	92.94 ± 0.16	89.00 ± 0.14	103.20 ± 0.50	99.14 ± 0.21
	Chroma (C*)	13.91 ± 0.05	18.12 ± 0.11	16.08 ± 0.11	14.89 ± 0.41	14.48 ± 0.27
Turbidity stability (% NTU)		6.87 ± 0.55 a	5.77 ± 0.11 ab	4.35 ± 0.36 b	6.72 ± 0.45 a	9.65 ± 0.91 c
Dry matter (%)		12.51 ± 0.01 c	11.15 ± 0.05 b	10.98 ± 0.01 a	11.19 ± 0.03 b	10.87 ± 0.03 a
Soluble solid (°Brix)		11.4 ± 0.0 a	10.2 ± 0.1 b	10.1 ± 0.0 b	10.2 ± 0.1 b	10.1 ± 0.0 b
Total acidity (g malic acid/100 mL)		1.38 ± 0.00 c	1.25 ± 0.00 ab	1.22 ± 0.00 ab	1.27 ± 0.03 b	1.21 ± 0.02 a
pH		3.03 ± 0.01 c	3.13 ± 0.01 ab	3.17 ± 0.03 b	3.08 ± 0.02 ac	3.14 ± 0.01 ab
Vitamin C content (mg/100 mL)		96.15 ± 2.88 a	98.33 ± 1.97 a	78.61 ± 1.57 c	89.54 ± 1.34 b	82.09 ± 1.64 c
Osmosis (mOsm/kg H_2_O)		785.5 ± 3.5 a	638.0 ± 55.2 a	666.0 ± 20.0 b	636.0 ± 24.0 a	631.5 ± 64.4 a
Energy Value (kcal/100 mL)		42.78 ± 0.94 a	39.03 ± 1.02 b	39.53 ± 0.59 b	34.44 ± 0.23 c	34.99 ± 0.77 c

* full product characteristics in Table 1; data are given as mean ± standard deviation (SD) (*n* = 2/3). Mean values within a column with different letters (a–d) are significantly different (homogenous groups) at *p* ≤ 0.05.

**Table 3:** Sugar and sugar alcohols content of obtained functional beverages.

Sugar Content (g/100 mL)	100% Pear–Flowering Quince Juice (0)	No. of Product *			
		1	2	3	4
Fructose	6.80 ± 0.17 a	5.96 ± 0.16 b	5.79 ± 0.15 b	6.00 ± 0.18 b	5.73 ± 0.12 b
Sorbitol	2.32 ± 0.04 a	1.99 ± 0.03 b	2.00 ± 0.04 b	2.03 ± 0.05 b	1.97 ± 0.02 b
Glucose	1.70 ± 0.02 a	1.48 ± 0.01 b	1.50 ± 0.03 b	1.51 ± 0.02 b	1.45 ± 0.04 b
Sucrose	0.86 ± 0.01 a	0.50 ± 0.00 c	0.49 ± 0.01 c	0.62 ± 0.01 b	0.64 ± 0.01 b
Total	11.68 ± 0.24 a	9.93 ± 0.20 b	9.78 ± 0.23 b	10.16 ± 0.26 b	9.79 ± 0.20 b

* full product characteristics in Table 1; data are given as mean ± SD (*n* = 3). Mean values within a column with different letters (a–c) are significantly different (homogenous groups) at *p* ≤ 0.05.

**Table 4:** Mineral content of obtained functional beverages.

Minerals Content (mg/100 mL)	100% Pear–Flowering Quince Juice (0)	No. of Product *				Recommended Dietary Allowance (mg/day)
		1	2	3	4	
Ca	3.93 ± 0.04 e	6.38 ± 0.02 b	8.23 ± 0.00 a	4.53 ± 0.00 d	5.76 ± 0.37 c	800 **/1000 ***
Na	0.91 ± 0.02 a	0.70 ± 0.11 a	0.74 ± 0.10 a	0.68 ± 0.12 a	0.73 ± 0.16 a	2000/1500
K	7.07 ± 0.25 ab	7.77 ± 0.29 a	6.30 ± 0.27 c	6.81 ± 0.24 bc	7.46 ± 0.28 a	2000/4700
Mg	0.29 ± 0.02 d	0.37 ± 0.03 d	10.43 ± 0.03 a	7.86 ± 0.06 c	9.26 ± 0.04 b	375/310–420
Fe	0.28 ± 0.03 c	0.31 ± 0.07 c	0.54 ± 0.03 b	0.27 ± 0.03 c	4.96 ± 0.39 a	14/8–18
Zn	nd	0.03 ± 0.00 a	nd	0.01 ± 0.01 b	0.02 ± 0.01 ab	10/11
Total	12.47 ± 0.36 e	15.54 ± 0.52 d	26.23 ± 0.43 b	20.16 ± 0.46 c	28.19 ± 1.25 a	

* full product characteristics in Table 1; data are given as mean ± SD (*n* = 3). Mean values within a column with different letters (a–e) are significantly different (homogenous groups) at *p* ≤ 0.05; nd—not detected; ** European Union; *** USA.

**Table 5:** Polyphenolic content of obtained functional beverages, and their bioavailability and bioaccessibility.

Polyphenolic Content (mg/100 mL)	100% Pear–Flowering Quince Juice (0)	No. of Product *			
		1	2	3	4
	in fresh product				
Phenolic acids	8.28 ± 0.14 d	14.92 ± 0.24 b	17.04 ± 0.51 a	7.44 ± 0.22 e	11.74 ± 0.35 c
Flavonols	2.13 ± 0.06 e	13.41 ± 0.20 b	24.83 ± 0.74 a	6.89 ± 0.21 d	10.66 ± 0.21 c
Flavan-3-ols (monomeric & dimeric)	81.96 ± 1.46 e	90.25 ± 1.70 c	167.41 ± 2.02 a	87.34 ± 1.62 d	101.90 ± 2.06 b
Polymeric procyanidins	402.48 ± 9.07 a	388.70 ± 8.66 b	369.90 ± 7.10 c	410.48 ± 9.31 a	403.55 ± 9.11 a
Total	494.85 ± 10.73 c	507.28 ± 10.80 bc	579.18 ± 10.37 a	512.15 ± 11.36 bc	527.85 ± 11.73 b
	bioaccessibility				
Phenolic acids	0.58 (7.00% **)	2.16 (14.48%)	3.08 (18.08%)	0.51 (6.85%)	1.50 (12.78%)
Flavonols	nd ***	4.36 (32.51%)	6.44 (25.94%)	0.33 (4.79%)	2.11 (19.79%)
Flavan-3-ols (monomeric & dimeric)	9.73 (11.87%)	5.23 (5.80%)	17.37 (10.38%)	5.05 (5.78%)	7.06 (6.93%)
Polymeric procyanidins	30.33 (7.54%)	23.89 (6.15%)	29.12 (7.87%)	23.38 (5.70%)	36.93 (9.15%)
Total	40.64 (8.21%)	35.64 (6.63%)	56.01 (9.67%)	29.27 (5.72%)	47.06 (9.02%)
	bioavailability				
Phenolic acids	nd	0.64 (29.62%)	0.92 (29.87%)	nd	nd
Flavonols	nd	0.91 (20.87%)	1.19 (18.48%)	nd	0.27 (12.80%)
Flavan-3-ols (monomeric & dimeric)	1.52 (15.62% ****)	1.24 (23.71%)	4.14 (23.83%)	0.89 (17.62%)	1.86 (26.35%)
Polymeric procyanidins	nd	nd	nd	nd	nd
Total	1.52 (3.74%)	2.79 (7.83%)	6.25 (11.16%)	0.89 (3.04%)	2.13 (4.53)

* full product characteristics in Table 1; data are given as mean ± SD (*n* = 3). Mean values within a column with different letters (a–e) are significantly different (homogenous groups) at *p* ≤ 0.05; ** % of bioaccessibility compared to the initial amount; *** nd—not detected; **** % of bioavailability compared to the bioaccessibility amount.

**Table 6:** Inhibition effects of obtained functional beverages.

Kind of Effect	Inhibition Effect IC50 (mg/mL)	100% Pear–Flowering Quince Juice (0)	No. of Product *			
			1	2	3	4
anti-diabetic	α-amylase	0.500^0.999^ ** e	0.526^0.999^ c	0.366^0.942^ a	0.518^0.999^ d	0.572^1.000^ b
	α-glucosidase	<0.020^0.999^ a	0.093^0.747^ b	1.523^0.798^ d	0.971^0.992^ c	1.495^0.999^ d
anti-obesity	pancreatic lipase	0.050^0.995^ a	0.057^0.991^ b	0.063^0.991^ c	0.056^0.954^ b	0.057^0.998^ b
anti-inflammatory	LOX-15	0.153^0.995^ b	0.149^0.971^ b	0.295^0.746^ d	0.179^0.911^ c	0.111^0.991^ a

* Full product characteristics in Table 1; ** coefficient of determination; data are given as mean ± SD (*n* = 3). Mean values within a row with different letters (a–e) are significantly different (homogenous groups) at *p* ≤ 0.05.

## Data Availability

The original contributions presented in this study are included in the article material. Further inquiries can be directed to the corresponding author.

## Abbreviations

SD	standard deviation
AAS	atomic absorption spectrometry
UPLC-PDA-FL	ultra performance liquid chromatography with photodiode array and fluorescence detectors
IC_50_	half maximal inhibitory concentration
ABTS	2,2′-azino-bis(3-ethylbenzothiazoline-6-sulfonic acid)
FRAP	the ferric reducing ability of plasma
ORAC	the oxygen radical absorbance capacity
LOX-15	15-Lipoxygenase
